# A case of cerebral venous sinus thrombosis associated with Crohn’s disease: dilemma in management

**DOI:** 10.1093/gastro/gou079

**Published:** 2014-11-10

**Authors:** Younghoon Kwon, Ryan J Koene, Yeilim Cho

**Affiliations:** ^1^St. Joseph’s Hospital, HealthEast Care System, Saint Paul, Minnesota, USA and; ^2^Department of Medicine, University of Minnesota, Minneapolis, Minnesota, USA

**Keywords:** cerebral venous sinus thrombosis, Crohn’s disease, anticoagulation, mercaptopurine, warfarin

## Abstract

Inflammatory bowel disease (IBD) is known to increase the risk of venous thromboembolism. Cerebral venous sinus thrombosis (CVST) is a rare but important complication of IBD. Timely diagnosis, particularly in younger patients, requires a high level of suspicion in order to prevent potentially devastating complications such as hemorrhage or venous infarction. The paper presents a 44-year-old Caucasian woman with a previous history of Crohn’s disease and deep venous thrombosis. Magnetic resonance imaging confirmed the diagnosis of CVST. Achieving therapeutic anticoagulation with warfarin was difficult, due to presumed pharmacological interaction between warfarin and 6-mercaptopurine. Clinicians should have a high index of suspicion for CVST when a patient with Crohn’s disease presents with acute headache, and be aware of challenges related to medical management.

## Introduction

Inflammatory bowel disease (IBD) is an autoimmune disorder of unknown etiology, which primarily involves the gastrointestinal tract. Crohn's disease and ulcerative colitis are the two main subtypes. Increased risk of venous thrombosis in IBD has been well described, usually within the deep veins of the legs or the pulmonary circulation [1]. We report a case of a female patient with active Crohn's disease, complicated by acute cerebral venous sinus thrombosis (CVST).

## Case presentation

**Figure 1.**
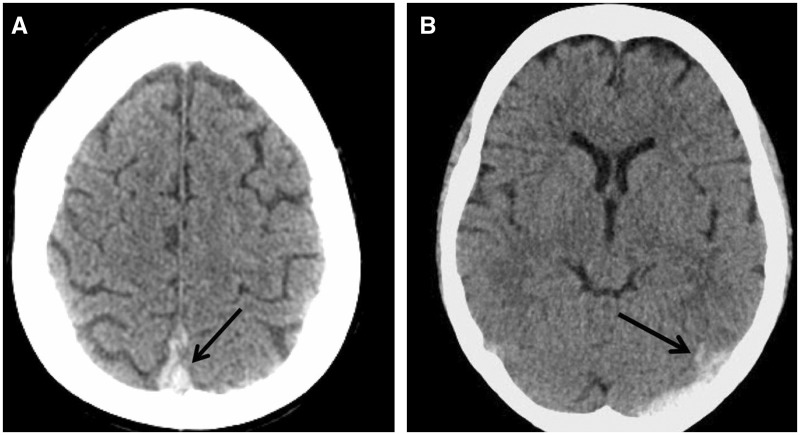
Computed tomography of the head on admission, showing hyperdensity within the superior sagittal sinus (**A**) and the left transverse sinus (**B**).

**Figure 2.**
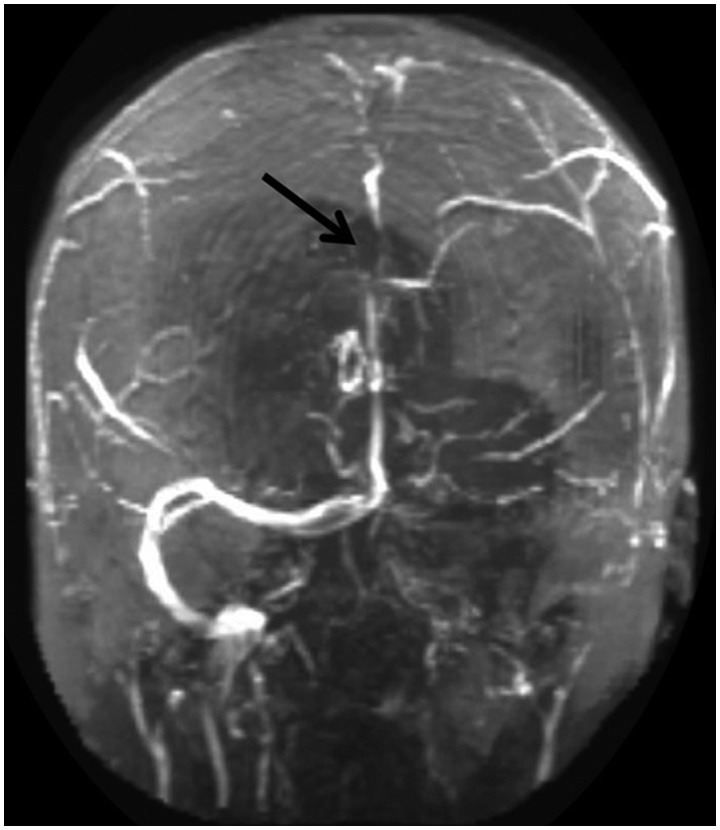
Magnetic resonance imaging with venography on admission, confirming the intraluminal thrombosis of the sagittal (arrow), transverse, and sigmoid sinuses, and extending into the cranial portion of the internal jugular vein.

A 44-year-old Caucasian woman with a history of Crohn's disease and deep vein thrombosis (DVT) was admitted to our hospital with a five-day history of a severe, throbbing and generalized headache. She had associated nausea, vomiting and photophobia. Her Crohn's disease history was notable for multiple bowel resections, including one within the past month. Home medications included 100 mg of 6-mercaptopurine (6-MP) once daily for Crohn's disease and an estradiol patch for menopausal symptoms. The latter was discontinued upon admission. Neurological examination showed mild meningismus upon neck flexion, with no other focal abnormalities. Computerized tomography (CT) of the head demonstrated a hyperdensity within the superior sagittal and left transverse sinuses, without parenchymal changes, suggesting dural sinus thrombosis (Figure 1). Subsequent magnetic resonance imaging with venography (MRI-MRV) confirmed an intraluminal thrombosis involving the sagittal, transverse, and sigmoid sinuses, and extending into the cranial portion of the internal jugular vein (Figure 2). Other abnormalities included an elevated platelet count of 501 x 10^9^/L and an increased C-reactive protein of 9.1 mg/L. Hypercoagulability assays showed heterozygosity for factor V Leiden (FVL) but no mutations in the prothrombin (G20210A) and methylenetetrahydrofolate reductase (C677T) genes. Thrombophilic profiling including protein C, S, antithrombin III activity, homocysteine and antiphospholipid antibodies was normal.

**Figure 3.**
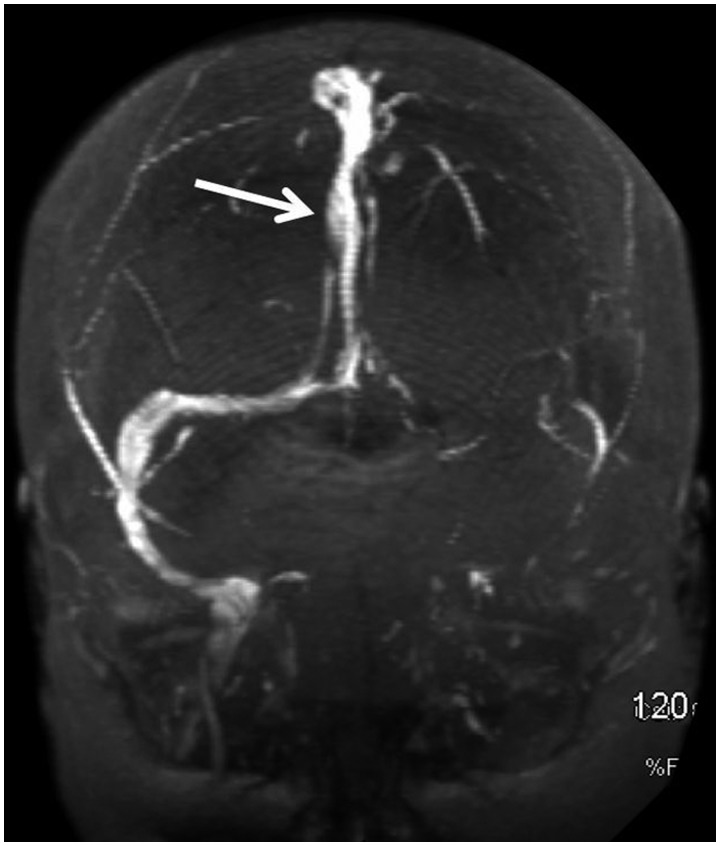
Magnetic resonance imaging with venography prior to discharge, demonstrating marked recanalization and improved flow signal (arrow).

After confirming CVST, the patient was started on i.v. heparin, followed by simultaneous administration of oral warfarin as a bridging therapy. Therapeutic international normalized ratio (INR) levels were difficult to achieve, requiring doses of warfarin as high as 20 mg daily. Although initially alert and oriented, the patient’s mental status declined rapidly, attributed to both high doses of opioid medications and increased intracranial pressure. However, withholding analgesics failed to improve her mental status. Despite absence of herniation on a repeat CT—given the clinical deterioration (worst Glasgow coma scale was 8) and significant burden of thrombosis on imaging while receiving i.v. and oral anticoagulation—endovascular intervention with direct intra-arterial i.v. tissue plasminogen activator (tPA) was attempted. Due to a lack of improvement in flow, direct mechanical thrombectomy was pursued. This resulted in considerable clot removal and improvement of flow. No complications were associated with these procedures. Within hours, the patient showed marked improvement in both mental status and severity of headache. MRV prior to discharge demonstrated marked recanalization and improved flow signal (Figure 3).

## Discussion

Cases of CVST associated with Crohn's disease have been rare in comparison to ulcerative colitis (Table 1) [2–6]. Although this may be simply due to underreporting of the cases, it probably reflects a higher overall incidence of thromboembolism in ulcerative colitis than Crohn's disease [7].

**Table 1.** Reported cases of cerebral venous sinus thrombosis in Crohn’s disease

Author	Year	Age/sex	Presenting symptoms	Time to diagnosis	Hypercoagulability	Treatment of CVST	Clinical course
Al-Malik *et al.* [2]	2001	14/M	Headache, seizure	1 day	**Normal**: protein C or S activity; ATIII; lupus anticoagulant	No treatment	Complete resolution of symptoms
					**Not mentioned:** FVL; prothrombin gene (G20210A); MTHFR (C677T) gene; homocysteine		
Samal *et al.* [3]	2004	20/M	Headache, vomiting, seizure	3 days	**Normal**- Antiphospholipid antibody; homocysteine level	Warfarin	Partial resolution of symptoms
					**Not obtained**: all others		
Magg *et al.* [4]	2004	30/M	Headache, lower extremity weakness	1 day	**Abnormal:** FVL heterozygous mutation; reduced protein C or S activity; increased platelet count	Rheolytic thrombectomy	Death
					**Normal**: prothrombin gene; MTHFR genes		
					**Not mentioned**: homocysteine; antiphospholipid antibody		
Rosen *et al*. [5]	2007	7/M	Periorbital headache, blurry vision, vomiting	Unknown	**Abnormal:** MTHFR gene homozygous mutation; prothrombin gene heterozygous mutation	Low molecular weight heparin	Complete resolution of symptoms
					**Normal:** FVL; protein C or S activity; ATIII; lupus anticoagulant		
					**Not mentioned:** homocysteine; anticardiolipin antibody		
Targosz-Gajniak *et al.* [6]	2010	31/M	Seizure	0 days	**Abnormal:** Increased platelet count	Osmotic agents	Complete resolution of symptoms
					**Not mentioned:** All others.		
Present case	2014	44/F	Headache, vomiting	5 days	**Abnormal:** FVL: heterozygous mutation; increased platelet	i.v. heparin, local tPA, mechanical thrombectomy, warfarin	Partial resolution of symptoms
					**Normal:** prothrombin gene; MTHFR genes; lupus anticoagulant; anticardiolipin antibody; homocysteine; ATIII; protein C or S activity		

ATIII = antithrombin; CVST = cerebral venous sinus thrombosis; FVL = Factor V Leiden; MTHFR = methylenetetrahydrofolate reductase; tPA = tissue plasminogen activator

### Pathophysiology

Based on the evidence of a close link between the coagulatory and immune systems in the pathogenesis of IBD, there has been speculation that the prevalence of the inherited risk of thrombophilia is higher for IBD [8]. Available studies, however, have largely shown a similar frequency of abnormal inherited prothrombotic factors; in particular, the incidence of the FVL mutation in thrombotic patients with IBD is similar to that in patients without IBD at about 14–15% [9]. Although it is unclear to what extent FVL increases the risk of thrombosis in IBD patients, it is plausible to assume a comparable increase (5–7 times increased relative risk in heterozygous mutations) to that seen in non-IBD patients. Consequently, hypercoagulability work-up is generally recommended. In our patient, the heterozygous FVL mutation probably compounded several acquired risk factors of venous thrombosis, including post-operative immobile status, thrombocytosis, and most importantly, the active state of Crohn's disease.

### Diagnosis and management

CVST typically affects a younger age range. Diagnosis is often challenging and requires a high index of suspicion because of varied clinical presentation and symptoms that can evolve over periods ranging from hours to a few weeks. Headache is the most common presenting symptom; in addition, seizures, focal neurological deficits and other signs of elevated intracranial pressure (ICP) may provide clues. CT can be a useful first diagnostic tool in ruling out other structural lesions including hemorrhage, but CT venogram or MRI-MRV is the diagnostic examination of choice to confirm CVST [10]. The most commonly employed treatment strategy known to be effective in improving outcomes is anticoagulation using i.v. heparin or subcutaneous low-molecular-weight heparin until the patient is stabilized, followed by oral anticoagulation for 3–6 months [11]. Lifelong anticoagulation could be considered for recurrent events in those with permanent risk factors. Anticoagulation alone may not be sufficient to improve the clinical condition when the clot burden is significant. If clinical deterioration continues despite anticoagulation, or if a patient has elevated ICP that increases despite other management approaches, more advanced techniques, such as direct thrombolytic therapy or endovascular treatment, may be considered as a salvage strategy, as illustrated in our case. There are no guideline recommendations discussing the utility of oral anticoagulation after successful thrombolytic or endovascular therapy, although several centers do report the initiation or continuation of oral anticoagulation [12].

### Unusual challenges in Crohn's disease

Our case highlights important aspects relevant to the medical management of venous thromboembolism—particularly CVST—in patients with Crohn's disease. Firstly, achieving therapeutic anticoagulation with an oral anticoagulant—a mainstay long-term treatment for CVST—can be potentially challenging. Our patient showed marked resistance to oral warfarin therapy, requiring an unusually high dose to maintain stable therapeutic levels. We attribute this mainly to a drug interaction that has been previously reported between warfarin and 6-MP [13, 14]. 6-MP is an immune modulating agent and an active metabolite of azathioprine, and is often used for patients who are refractory to first-line agents for Crohn's disease, such as glucocorticoids. Two cases have reported a doubled warfarin dose requirement when 6-MP was used concurrently [13, 14]. While the underlying mechanism of this interaction has not been fully elucidated, enhancement of prothrombin (factor X) and factor II activity has been suggested, based on an animal study that demonstrated a reduction in the prothrombin time and higher prothrombin complex activity when rats were given warfarin intravenously and 6-MP orally [15]. It is worth noting that similar interaction has been reported with azathioprine, another commonly used immune-modulator in Crohn's disease [16, 17]. Thus clinicians should closely monitor INR when oral anticoagulation therapy is administered, particularly in the setting of immune modulators.

The rather exaggerated warfarin resistance seen in our case may be partially attributed to reduced absorption of warfarin in the small bowel, due to loss of effective surface area from multiple resections in the past (i.e. ‘short gut' syndrome). Lastly, although absent in our patient, active gastrointestinal bleeding—not uncommon in the active stage of Crohn's disease—is a contraindication to therapeutic anticoagulation. Conversely, concurrent intracranial hemorrhage related to CVST is not a contraindication for anticoagulation therapy [18].

## Conclusions

Prompt diagnosis and timely treatment of CVST can be facilitated by a high level of suspicion when a patient with Crohn's disease presents with headache or other neurological symptoms. Clinicians should be aware of potential challenges to medical management that are unique to Crohn's disease.
